# Soluble endoglin as a marker of clinically significant portal hypertension in patients with cirrhosis

**DOI:** 10.1038/s41598-026-51523-w

**Published:** 2026-05-02

**Authors:** Ivone Cristina Igreja Sa, Vaclav Smid, Petra Fikrova, Jana Urbanková Rathouska, Ivana Nemeckova, Katarina Tripska, Adela Diepoltova, SeyedehNiloufar Mohammadi, Ctirad Andrys, Jaromir Petrtyl, Radan Bruha, Libor Vitek, Petr Nachtigal

**Affiliations:** 1 Department of Biological and Medical Sciences, Faculty of Pharmacy in Hradec Kralove, Charles University, Heyrovskeho 1203, 500 05 Hradec Kralove, Czech Republic; 2 Department of Clinical Microbiology, Faculty of Medicine in Hradec Kralove, Charles University, Simkova 870, Hradec Kralove, 500 03 Charles, Czech Republic; 3 st Faculty of Medicine and General University Hospital in Prague, Charles University, U Nemocnice 499/2, Prague, 128 08 Czech Republic; 4 Department of Immunology and Allergology, Faculty of Medicine in Hradec Kralove, University Hospital Hradec Kralove, Charles University, Sokolska 581, 500 05 Hradec Kralove, Czech Republic; 5 Institute of Medical Biochemistry and Laboratory Diagnostics 1 st Faculty of Medicine, General University Hospital in Prague, Charles University, Katerinska 32, Prague, 121 08 Czech Republic

**Keywords:** soluble endoglin, portal hypertension, metabolic dysfunction-associated steatotic liver disease, alcohol-related liver disease, fibrosis, Biomarkers, Diseases, Gastroenterology, Medical research

## Abstract

Portal hypertension (PH) is one of the major complications of liver cirrhosis, traditionally assessed using invasive methods such as the hepatic venous pressure gradient (HVPG). Soluble endoglin (sENG), a marker of endothelial dysfunction and fibrosis, has been proposed as a non-invasive biomarker of various liver diseases. This study aimed to evaluate serum sENG concentrations in cirrhotic patients with PH and investigate its relationship with PH severity, alcohol consumption, and smoking. Serum concentrations of sENG were measured in clinically well-examined patients with liver cirrhosis (*n* = 60, age range 24–82 years) with PH classified as mild, moderate, or severe according to the HVPG values measured invasively using the classical wedge technique. sENG concentrations were also compared to healthy controls (*n* = 54). Liver enzyme activities, alcohol consumption history, and smoking habits were also recorded to assess their association with sENG. sENG concentrations were significantly higher in patients with PH compared to healthy controls (6.31; 5.14–7.30 vs. 3.70; 3.24–4.20 ng/mL, *p* < 0.001) but did not correlate with the severity of HVPG-diagnosed portal hypertension. A moderately significant correlation was observed between sENG concentrations and GGT activities (*p* < 0.001). Alcohol consumption, but not smoking, was associated with higher serum sENG concentrations (*p* < 0.01). Based on our results, sENG appears to be a non-invasive marker of endothelial dysfunction/fibrosis in cirrhotic patients with PH, particularly in alcohol-related liver disease. Although it does not reflect PH severity and thus cannot be used as a diagnostic tool, it has the potential for early disease detection and risk prediction as a screening component in non-invasive approaches in clinical hepatology.

## Introduction

The prevalence of chronic liver diseases is high and increasing throughout the world, representing one of the major causes of morbidity and mortality worldwide^1^. Chronic liver diseases usually arise from chronic inflammation of various origins, which may lead to progression to liver fibrosis and subsequently liver cirrhosis. The Global Burden of Disease 2023 study reported that 1.26 million people died from cirrhosis and other chronic liver diseases in 2019, marking a 13% increase since 1990^2^. The total worldwide prevalence has been estimated to be around 1%, with regional variation depending on the occurrence of viral hepatitis, metabolic syndrome, and alcohol consumption^3^.

Advanced liver cirrhosis is associated with portal hypertension (PH), leading to the development of esophageal and gastric varices, portal hypertensive gastropathy, ascites, spontaneous bacterial peritonitis, hepatorenal syndrome, hepatopulmonary/portopulmonary syndromes, and hepatic encephalopathy. Together with hepatocellular carcinoma, these complications represent the main causes of death in patients with liver cirrhosis^4^.

Measurement of the hepatic venous pressure gradient (HVPG) is considered the gold standard for diagnosing and evaluating the severity of PH and estimating the risk of variceal bleeding. Measurement of HVPG is a reliable method to quantify portal pressure and predict the occurrence of complications, as well as to monitor the effectiveness of therapy. Although HVPG is generally safe and well-tolerated^5^, this invasive procedure is not routinely available and is restricted to tertiary/specialized medical centers. Furthermore, it does not enable long-term monitoring. Together with esophagogastroduodenoscopy, which can reliably demonstrate the presence of varices and the risk of bleeding, both procedures are invasive and often not well accepted by patients who may refuse further follow-up. Another disadvantage is the high costs of these procedures. Thus, new non-invasive methods for the assessment of PH in patients with liver cirrhosis are necessary.

Endoglin (ENG, CD105) is a 180 kDa transmembrane glycoprotein considered a co-receptor for ligands of the TGFβ superfamily. There are two different forms of endoglin, specifically a membrane ENG expressed by various cells (endothelial cells, macrophages and smooth muscle cells) and a soluble endoglin (sENG) that circulates in serum or cell culture medium^6^. sENG is the N-terminal cleavage product of the extracellular domain of ENG formed by the activity of matrix metalloproteinases^7–9^ that is released into circulation. sENG can be detected and used as a biomarker in various cardiovascular and metabolic disorders, such as preeclampsia^10^, hypercholesterolemia^11^, familial hypercholesterolemia^12^, atherosclerosis^13^, arterial hypertension^14^, septic shock disease^15^, and liver alterations^16^. Interestingly, serum concentrations of sENG have recently been shown to be related to smoking^17^. Moreover, increased serum concentrations of sENG were demonstrated during the development of early aortic endothelial dysfunction^18^ and liver sinusoidal endothelial dysfunction during progression of metabolic dysfunction-associated steatohepatitis (MASH)^16^, and correlated with total serum cholesterol concentrations and the progression of atherosclerosis in vivo^19^. These data suggest its importance in the development of endothelial dysfunction in various parts of the circulation and blood vessels. In addition, it was demonstrated that high serum concentrations of sENG aggravate aortic endothelial dysfunction^20^ and endothelial inflammation^21^, suggesting their potentially harmful effects on the vascular endothelium.

Importantly, previous studies found that sENG can significantly alter bile acid (BA) metabolomics in healthy mice and mice suffering from MASH^22,23^. Moreover, sENG has been reported to be a biomarker of different liver-related diseases, such as cystic fibrosis-associated liver diseases^24^, biliary atresia^25^, MASH^26^, intrahepatic cholestasis^22^, and liver fibrosis^27^. Prystupa et al.. showed that sENG concentrations differed significantly between the control group and patients with Child–Pugh stage B and C cirrhosis. Moreover, they demonstrated that bilirubin concentration, insulin resistance, and duration of alcohol abuse were independent factors with respect to sENG concentration^27^.

Therefore, in this study, we hypothesized that serum concentrations of sENG would be higher in patients with PH and could possibly reflect the severity of the PH. Furthermore, our objective was to evaluate the relationship of alcohol consumption and smoking with serum sENG concentrations in these patients.

## Methods

A group of 60 patients (age range 24–82 years, M:F ratio = 41:19) with clinically confirmed liver cirrhosis was included in the study. They were recruited from patients of the 4th Department of Internal Medicine (Gastroenterology and Hepatology) of the General University Hospital in Prague between 2003 and 2019. In each patient, all the examinations mentioned below were performed during one or two weeks. A group of 54 healthy controls (age range 21–53 years, M:F ratio = 29:25) was recruited from consecutive healthy blood donors from the General University Hospital Hradec Kralove. Inclusion criteria for the healthy subject group were the absence of any apparent liver disease (as evidenced by physiological values of standard liver enzyme activities), alcohol abuse, diabetes mellitus, or metabolic syndrome.

The study was carried out in full accordance with the Helsinki Declaration of 1975 as revised in 1983 and was approved by the Institutional Ethics Committee. Written informed consent for the future evaluation of blood samples for scientific purposes was obtained from all subjects at the time of hepatic vein catheterization.

Liver cirrhosis was diagnosed based on laboratory tests, clinical and ultrasound findings, and/or liver biopsies; the presence of PH was diagnosed by hepatic vein catheterization and HVPG measurement. Exclusion criteria were thrombosis of the portal vein, a history of transjugular intrahepatic portosystemic shunts, hepatocellular carcinoma, and/or diseases of the spleen.

Alcohol intake was assessed using a structured patient interview, with quantification based on self-reported average weekly consumption over the previous 12 months. Consumption exceeding 20 g/day for women and 30 g/day for men was considered clinically significant, in accordance with the European Association for the Study of the Liver (EASL) recommendations. In patients diagnosed with alcohol-related liver disease (ALD), the etiology was established by comprehensive patient interviews that confirmed a history of sustained excessive alcohol consumption or by consistent and long-term evidence documented within the patient’s medical records. In patients with metabolic dysfunction-associated steatotic liver disease (MASLD)/MASH etiology of liver cirrhosis, significant alcohol abuse was ruled out through the patient’s personal history, a self-reported questionnaire, stable gamma-glutamyltransferase (GGT) activities documented in the patient’s medical records, and determination of serum carbohydrate-deficient transferrin and/or urine ethyl-glucuronide concentrations, if needed.

Clinical, anthropometric, and laboratory parameters were recorded. Biochemical analyses were performed on an automatic analyzer (Modular Analyzer; Roche Diagnostics GmbH, Mannheim, Germany) using standard laboratory assays. The severity of liver disease was evaluated using the Child-Pugh score and the Model for End-Stage Liver Disease (MELD) score. All patients underwent standard abdominal ultrasound examination.

### HVPG measurement

Measurement of the HVPG was performed using the classical wedge technique^5^. After overnight fasting, the patients were transported to the catheterization room. Under local anesthesia, a 7 F catheter introducer was placed in the right jugular vein using the Seldinger technique. Under fluoroscopic control, a 7 F balloon-tipped catheter (B. Braun Melsungen AG, Melsungen, Germany) was advanced into the right hepatic vein in order to measure both the free hepatic venous pressure and the wedged hepatic venous pressure. All measurements were performed in triplicate using a continuous recording unit. HVPG was calculated as the difference between the wedged hepatic venous pressure and the free hepatic venous pressure. Clinically significant PH was defined according to the Baveno criteria with HVPG ≥ 10 mmHg^28^.

In addition to the clinical significance threshold, AASLD in its guidelines on PH risk stratification and management^29–31^, states the HVPG threshold of 12 mmHg, above which there is significant development of clinical complications, such as risk for variceal bleeding; and an HVPG > 20 mmHg, that correlates with worse clinical outcomes, such as poor control of acute variceal bleeding, higher risk of rebleeding, high risk of clinical decompensation and increased mortality^32,33^. Based on these thresholds, PH patients were classified into severity groups according to their HVPG results. In this study, HVPG values above clinically significant PH (10 mmHg) but below the clinical complication’s cutoff (12 mmHg) were classified as “mild” (10; 9.25–11 mm Hg), HVPG values above complication’s cutoff (12 mmHg) but below the cutoff predictor of worse clinical outcomes (20 mmHg) as “moderate” (14.5; 13-15.75 mmHg) and HVPG values above the predictor of worse disease outcomes (20 mmHg) as “severe” (22.5; 20–24 mm Hg).

### Measurement of sENG by ELISA

The concentrations of sENG were assessed in serum samples by sandwich enzyme-linked immunosorbent assay technique (ELISA) using the Quantikine Human Endoglin/CD105 ELISA kit (R&D Systems, MN, USA) according to the manufacturer’s instructions in undiluted samples. The sensitivity of the kit was 0.007 ng/mL. The absorbance values were measured at 450 nm for signal detection and 540 nm for background correction with a Multiskan RC ELISA reader (Thermo Fisher Scientific, MA, USA).

### Statistical analysis

Clinical and laboratory data are presented as median with interquartile range (IQR). On the basis of normality testing of the data, non-parametric tests were used in statistical analyses. Comparison between multiple independent groups was performed by Kruskal-Wallis, followed by post-hoc Dunn’s multiple comparison test. Direct between-group comparisons were carried out using a Mann-Whitney test. Correlations were assessed by the determination of Spearman’s coefficient. P-values < 0.05 were considered statistically significant. Statistical analyses were performed using the GraphPad Prism software version 10.4 (GraphPad Software Inc., San Diego, CA, USA).

## Results

### Patient cohort characteristics

A cohort of 60 PH patients with confirmed liver cirrhosis was included in this study and subsequently divided into groups according to PH severity. Patient characteristics are further summarized in Table 1. Upon comparison between the activity of liver enzymes, no significant difference was found between the three degrees of PH severity for ALT, ALP, or AST. Similarly, no statistically significant correlation was found between GGT or bilirubin and the severity of PH.

**Table 1:** Clinical and laboratory parameters of patients.

Parameters	Liver cirrhosis All (*n* = 60)	mild PH (*n* = 20)	moderate PH (*n* = 20)	severe PH (*n* = 20)
Age [years]	56.3 ± 12.7	52.6 ± 12.1	58.2 ± 13.4	58.1 ± 12.5
Male gender [%]	68.3	75.0	60.0	70.0
M: F ratio	41:19	15:5	12:8	14:6
Bilirubin [µmol/l]	26.8 ± 15.8	23.6 ± 12.6	26.0 ± 16.1	30.5 ± 18.0
ALT [µkat/L]	0.96 ± 1.29	1.38 ± 1.63	0.93 ± 1.43	0.56 ± 0.34
ALP [µkat/L]	2.08 ± 1.12	2.21 ± 1.33	1.84 ± 0.93	2.19 ± 1.07
AST [µkat/L]	1.18 ± 1.04	1.44 ± 1.12	1.17 ± 1.34	0.95 ± 0.43
GGT [µkat/L]	3.21 ± 3.07	4.52 ± 3.35	2.14 ± 1.88	2.96 ± 3.38
Albumin [g/L]	34.9 ± 7.2	39.6 ± 5.9	31.2 ± 7.9	33.6 ± 5.0
Thrombocytes [10^9^/L]	124.2 ± 80.6	140.5 ± 112.2	122.8 ± 76.6	109.4 ± 34.4
INR	1.27 ± 0.27	1.15 ± 0.19	1.24 ± 0.23	1.41 ± 0.31
HVPG [mm Hg]	15.4 ± 5.4	9.9 ± 1.0	14.2 ± 1.5	22.1 ± 2.3
Child-Pugh score Child-Pugh score A/B/C [%]	7 ± 2 40 / 52 / 8	6 ± 1 80 / 20 / 0	7 ± 2 50 / 40 / 10	7 ± 2 25 / 60 / 15
MELD score	9 ± 6	8 ± 4	9 ± 6	11 ± 6
Esophageal varices [%] (none/small/large)	28 / 36 / 36	39 / 28 / 33	35 / 40 / 25	10 / 40 / 50

Values expressed as mean ± SD.

*ALT*,* alanine aminotransferase; ALP*,* alkaline phosphatase; AST*,* aspartate aminotransferase; GGT*,* γ-glutamyl transferase; HVPG*,* hepatic venous pressure gradient; INR*,* international normalized ratio.*

### Serum concentrations of sENG are higher in patients with PH, but do not correlate with disease severity

**Fig. 1:**
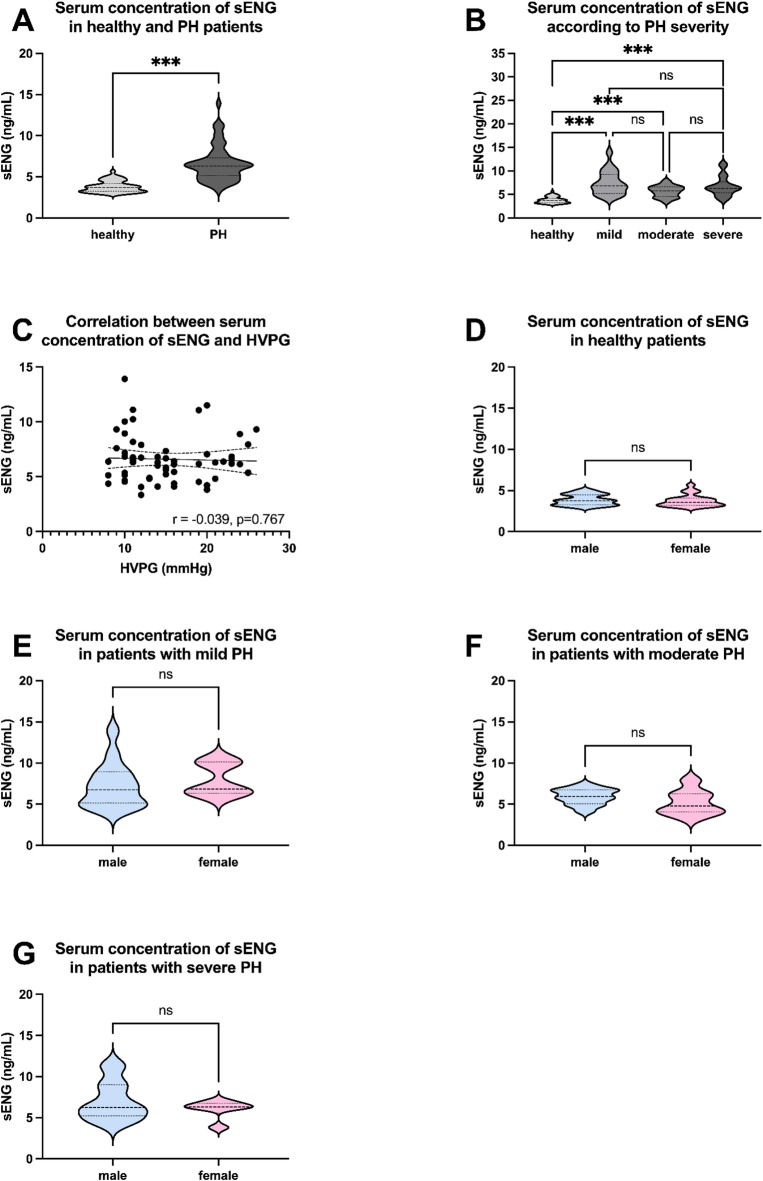
Serum concentrations of sENG in healthy controls and all patients with portal hypertension (PH) (A) and patients with different severities of PH (B). Correlation between serum concentration of sENG and HVPG (C). Comparison of sENG values between male and female control patients and patients within the same degree of PH (C, D, E, F). Data presented as median (IQR). Statistical analysis for multiple comparisons was performed using Kruskal-Wallis, followed by Dunn’s post-hoc test. Direct between-group comparisons were performed by the Mann-Whitney test. *** *p* < 0.001; ns, non-significant.

Serum concentrations of sENG were significantly higher in patients with PH compared to healthy controls (6.31; 5.14–7.30 vs. 3.70; 3.24–4.20 ng/mL) (Fig. 1A). In addition, serum concentrations of sENG in patients with PH were significantly higher in all groups compared to healthy controls, but similarly as liver enzymes, no significant difference was found concerning PH severity (6.80; 5.20–9.23, 5.73; 4.49–6.62 and 6.27; 5.36–7.72 ng/mL) in patients with mild, moderate and severe PH, respectively (Fig. 1B). Moreover, there was no significant correlation between sENG serum concentration and HVPG (Fig. 1C). Concentration of sENG was not related to sex since no difference was observed between male and female controls and patients within the same severity stage (Fig. 1D, E, F, G).

### Serum concentrations of sENG are higher in patients with PH who report alcohol consumption

**Fig. 2:**
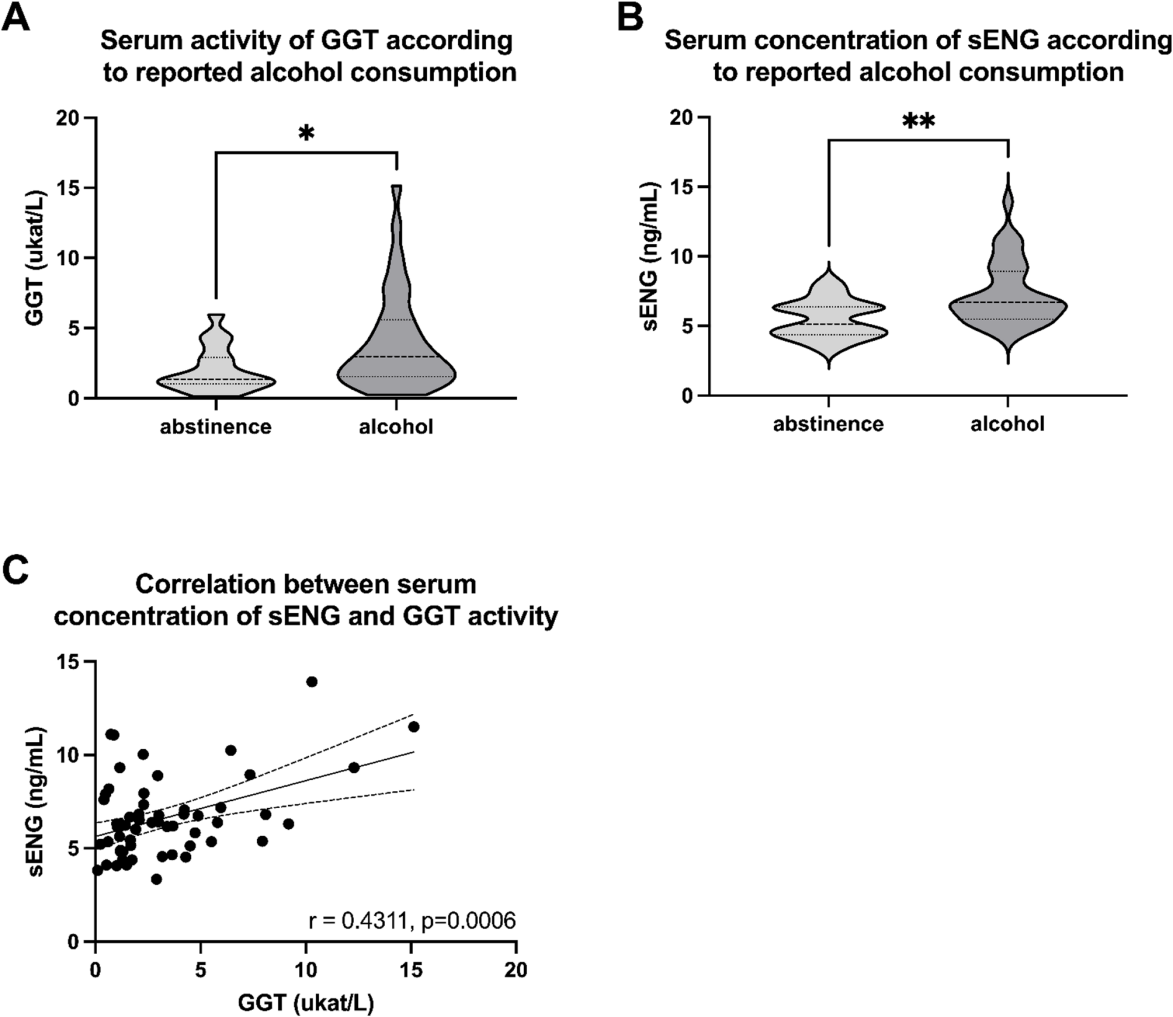
Relationship between GGT activities (A) and serum concentrations of sENG (B) in cirrhotic patients with PH according to alcohol consumption and correlation between serum concentrations of sENG and GGT activities (C). Data presented as median (IQR). * *p* < 0.05; ** *p* < 0.01. (C) Interpolation of sENG vs. GGT activities. The solid line corresponds to the linear regression line fitted to the data, while the dotted lines represent the 95% confidence interval of the regression line.

Cirrhotic patients with PH who were not abstaining from alcohol had higher GGT activities than PH patients who abstained from alcohol for more than 20 years (2.98; 1.54–5.59 vs. 1.35; 1.02–2.90 ukat/L) (Fig. 2A). The same relationship was also observed for serum concentrations of sENG. Indeed, alcohol consuming cirrhotic patients with PH had higher serum concentrations of sENG compared to cirrhotic abstainers (6.72; 5.49–8.93 vs. 5.14; 4.38–6.38 ng/mL, *p* < 0.01) (Fig. 2B).

A positive correlation was observed between the serum concentration of sENG and GGT activities (*r* = 0.4311, *p* < 0.001) (Fig. 2C). A multivariate linear regression analysis was performed adjusting for age, sex, MELD score, and alcohol consumption. The overall model was statistically significant (*p* = 0.002). The results show that GGT remains significantly associated with sEng after the adjustments mentioned (β = 0.197, *p* = 0.032), indicating that this association is independent of the above potential confounders. Age did not contribute significantly to sEng concentrations (*p* = 0.320), suggesting that the observed age difference between the groups does not confound the GGT–sEng relationship. The same was observed for the MELD score (*p* = 0.964) and sex (*p* = 0.773), which were not significant predictors. However, alcohol consumption was also an independent predictor (β = 1.41, *p* = 0.011) for sEng. Multicollinearity was low (all VIF < 1.27).

### Alcohol consumption is associated with increased serum concentrations of sENG, whereas smoking does not have any effect

**Fig. 3:**
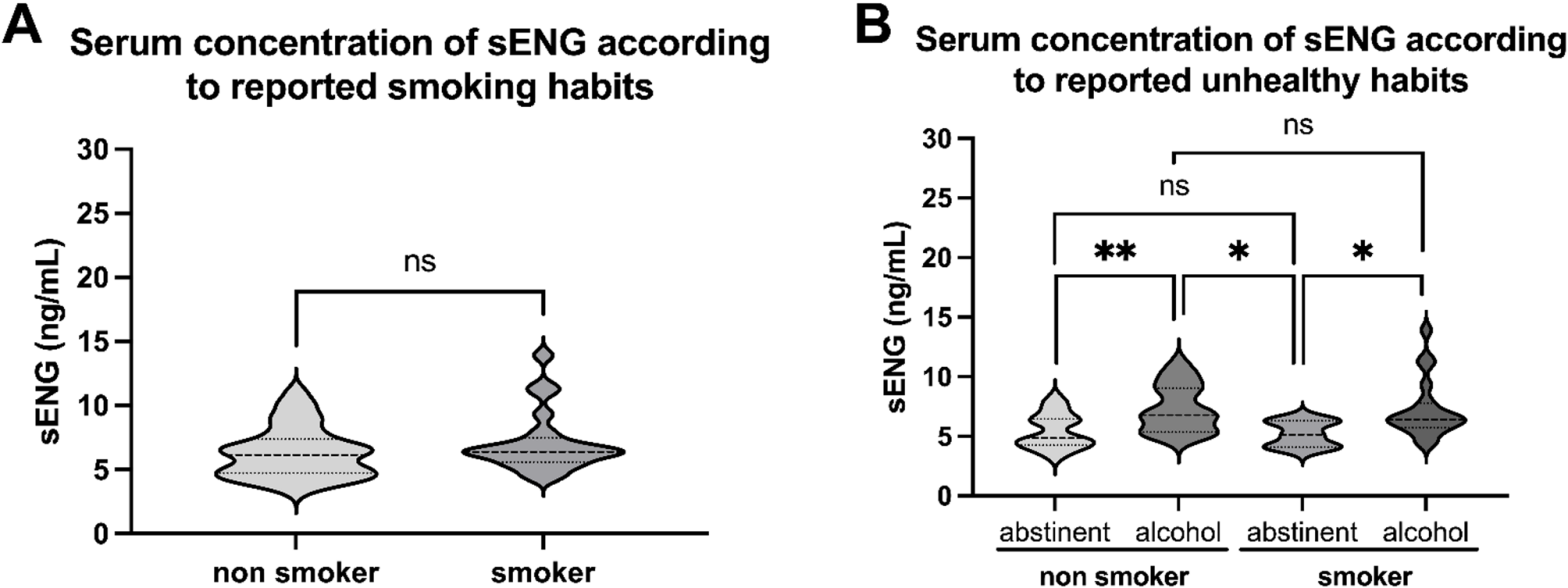
Serum concentrations of sENG in cirrhotic patients with PH according to the smoking status (A) and unhealthy habits (B). Data presented as median (IQR). Direct between-group comparisons were performed by the Mann-Whitney test. * *p* < 0.05, ** *p* < 0.01; ns, non-significant.

The evaluation of serum concentrations of sENG according to smoking status in cirrhotic patients with PH did not show significant differences between smokers and non-smokers (6.38; 5.59–7.49 vs. 6.15; 4.74–7.40 ng/mL, *p* = 0.215), regardless of alcohol consumption (Fig. 3A). When patients are categorized according to alcohol consumption and/or smoking habits we recorded no statistically significant difference between abstinent smokers and abstinent non-smokers (5.14; 4.09–6.31 vs. 4.86; 4.26–6.47 ng/mL, *p* = 0.780), as well as between smokers abusing alcohol and non-smokers abusing alcohol (6.40; 5.73–7.79 vs. 6.79; 5.37–9.04 ng/mL, *p* = 0.718) (Fig. 3B). However, significantly higher serum concentrations of sENG were observed in non-smoking alcohol abusers when compared to non-smoking alcohol abstainers (6.79; 5.37–9.04 vs. 4.86 4.26–6.47 ng/mL, *p* = 0.003), and in smoking alcohol abusers when compared to smoking alcohol abstainers (6.4; 5.73–7.79 vs. 5.14; 4.09–6.31 ng/mL, *p* = 0.035), as well as in a direct comparison between those patients who only consume alcohol and those who just smoke (6.79; 5.37–9.04 vs. 5.14; 4.09–6.31 ng/mL, *p* = 0.015) (Fig. 3B). Pearson’s correlation did not reveal any relationship between sENG and liver enzymes (ALT r(*n* = 60) = -0.071, *p* = 0.58; AST r(*n* = 60) = 0,086, *p* = 0.51; ALP r(*n* = 60) = 0,231, *p* = 0.07.

## Discussion

In this study, for the first time, we investigated the role of sENG as a possible non-invasive biomarker in patients with PH and liver cirrhosis. Our results showed that sENG serum concentrations were significantly higher in cirrhotic patients with PH compared to healthy controls, and in patients with PH and alcohol abuse compared to abstinent cirrhotic patients. Moreover, sENG concentrations increase significantly even in patients with mild portal hypertension, suggesting that sENG may serve as a sensitive marker of early-stage PH.

sENG is a circulating form of membrane ENG produced when the extracellular portion of membrane-bound ENG is cleaved by matrix metalloproteinases (MMP), especially MMP-14^34^. sENG plays a significant role in liver disorders, particularly in liver fibrogenesis, cirrhosis, hepatocellular carcinoma, and MASH. sENG also serves as both a biomarker of disease severity and a modulator of critical metabolic processes within the liver^16,26,35^. In addition, higher concentrations of sENG were observed during the development of MASH and intrahepatic cholestasis, which were associated with liver sinusoidal endothelial dysfunction (LSED) and fibrosis development^16^. Based on these facts, we hypothesized that the serum concentrations of sENG could reflect the presence and severity of PH in humans and be related to alcohol abuse and smoking.

The results showed that sENG concentrations are significantly elevated in both male and female patients with PH but do not correlate with PH severity. The lack of correlation between sENG concentrations and HVPG or the severity of PH may be explained by the different pathophysiological processes these parameters reflect. While HVPG directly measures intrahepatic hemodynamic pressure, sENG is more likely a marker of systemic or liver LSED or fibrosis, as supported by previous studies linking elevated sENG to vascular injury and endothelial cell activation^36^. In this context, increased concentration of sENG in cirrhotic patients may indicate the presence of widespread endothelial dysfunction or possibly the development of fibrosis, rather than changes in the hepatic portal pressure specifically. Thus, sENG and HVPG represent distinct aspects of the disease process: HVPG provides a hemodynamic assessment of PH, whereas sENG may reflect earlier molecular alterations associated with endothelial damage and the fibrosis process. This could explain why its levels increase markedly and significantly even in the initial degrees of PH, while in more advanced degrees of PH (HVPG > 12 mm Hg), a further increase likely does not occur.

Interestingly, sENG release can be related to LSED or the liver fibrosis process. Indeed, sENG concentrations are increased during the development of endothelial dysfunction in humans^36^ and mice^18^. Moreover, sENG concentrations were increased in various situations with confirmed liver fibrosis^16,35^. Importantly, sENG was also shown to aggravate endothelial dysfunction^20^ and liver alteration^22,23^, suggesting potentially harmful effects of high concentrations of sENG on the liver. On the other hand, we cannot assess whether increased sENG concentrations in PH and liver cirrhosis patients are related to LSED, liver fibrosis, or both.

This interpretation is further supported by our observation that sENG concentration also does not correlate with the activities of the liver enzymes, which are commonly used to assess hepatocellular injury^37^. This lack of association suggests that sENG reflects a pathophysiological mechanism different from a simple liver cell necrosis or inflammation. Whereas ALT and AST are markers of hepatocellular damage, sENG may instead indicate endothelial activation, injury, or fibrogenic responses, processes that are not reflected by conventional liver function tests.

Another key finding of this study was the effect of alcohol consumption on sENG concentrations. Patients with PH with continued alcohol use had significantly higher concentrations of sENG and GGT activity compared to patients with PH who had abstained from alcohol for more than 20 years. GGT is an enzyme mainly produced by cells of the hepatobiliary tract, which has a microsomal isoform inducible by alcohol^38^. In our study, a moderate positive correlation was also observed between GGT and sENG, suggesting a potential link between alcohol-related liver injury and endothelial dysfunction/fibrosis, which is consistent with previous published studies in alcohol-induced endothelial injury^39^. These findings are in agreement with the known effects of alcohol in promoting oxidative stress and inflammation, which can enhance the cleavage and release of sENG from endothelial cells. A possible mechanism for our findings lies in the known premise that chronic alcohol intake can produce oxidative stress and inflammation^40^ that can also lead to MMP activation^41^. MMPs are also known to be increased/overexpressed in cirrhosis and fibrosis^42^, which can result in the cleavage and release of sENG. In addition, alcohol intake decreases nitric oxide production in liver sinusoidal endothelial cells, increases endothelin-1 production, which increases portal pressure and induces LSED^43^. Interestingly, LSED development is characterized by increased concentrations of sENG in the circulation^35^. Therefore, the GGT-sENG correlation supports this premise, since GGT is a marker of alcohol abuse, liver oxidative stress, and hepatobiliary injury^38^. The potential association between sENG concentrations and alcohol intake could be further supported by the determination of phosphatidylethanol (Peth), a blood biomarker reflecting alcohol use and allowing objective detection of intake for up to four weeks. The use of this marker would provide a more accurate picture of recent drinking than the self-report alone, supporting a better assessment of patients with steatotic liver disease^44^. When combined with questionnaires and clinical evaluation, PEth and possible sEng could help clarify discrepancies, monitor adherence over time, and better understand the contribution of alcohol to disease progression. Additionally, a multivariate linear regression analysis adjusting for age, sex, MELD score, and alcohol consumption showed that GGT remains significantly associated with sENG, indicating that this association is independent of these potential confounders.

Additionally, smoking status did not significantly affect sENG concentrations. Although some previous studies have suggested a relationship between smoking and both sENG^17^ and GGT^38^, our results indicate that in cirrhotic patients with PH, alcohol consumption has an impact on sENG concentrations whereas smoking does not; the effect of smoking may be masked by more dominant factors, such as alcohol abuse or fibrosis, or simply the alcohol consumption works as a trigger for sENG release in the circulation (supporting our hypothesis). Despite the need for a deeper understanding and evaluation of sENG concentrations in a larger cohort of PH patients (and possibly in cirrhotic patients with a broader range of hepatic fibrosis severity), the positive association of sENG with alcohol consumption reported in this study is of high clinical relevance. In fact, the stigma associated with alcohol consumption is real and represents a significant barrier in clinical research, not only negatively impacting the willingness of individuals to participate in studies, but also influencing the information they provide to the medical staff during anamnesis, due to shame or embarrassment, which could reduce the quality of care they receive^45^. Given the association between sENG and alcohol consumption, the assessment of sENG by routine blood tests may have the potential to be used as a proxy indicator or a predictive measure for chronic alcohol consumption, particularly in instances where alcohol consumption cannot be easily assessed (e.g., due to stigma, reporting bias, or other barriers to disclosure).

There are possible limitations of this study. In the first place, the study was not designed with longitudinal follow-up, and clinical outcome data were not systematically available. Therefore, the prognostic value of sENG for liver-related events could not be assessed. This should be considered a limitation, and future prospective studies are needed to clarify the predictive role of sENG. Another potential limitation of our study is the relatively long recruitment period (2003–2019), during which changes in clinical practice may have occurred, including changes in the use of non-selective beta-blockers and in the general management of portal hypertension. Although treatment strategies followed the standard of care at the time of inclusion, and the present analysis focused primarily on pathophysiological and hemodynamic associations rather than treatment effects, we cannot fully rule out the possibility that temporal changes in patient management may have influenced the cohort characteristics. This should be considered when interpreting the results.

In conclusion, this study supports the role of sENG as a marker of endothelial dysfunction and/or fibrosis in patients with PH and liver cirrhosis. Although sENG concentrations were significantly higher in cirrhotic patients than in healthy controls, they did not distinguish between different degrees of PH severity. The measurement of HVPG, although invasive, is the gold standard and the most reliable method for the classification of PH according to its severity; so far, no other biochemical parameters have shown a correlation with it. Therefore, it is currently not possible to use non-invasive methods instead. Importantly, alcohol consumption, but not smoking, was associated with higher sENG concentrations, highlighting its potential value in identifying alcohol-related liver injury.

Although more longitudinal and mechanistic studies are needed, our data suggest that in clinical practice, sENG can help identify the presence of PH or alcohol-related endothelial dysfunction/fibrosis, particularly when used alongside other established markers, potentially enhancing early detection or monitoring of systemic vascular involvement and risk stratification in cirrhotic patients. However, its diagnostic utility requires further validation.

## Data Availability

The original contributions of this study are detailed in the article; further inquiries can be directed to the corresponding author.

## Abbreviations

ALD: alcohol–related liver disease
ENG: endoglin
HVPG: hepatic venous pressure gradient
LSED: liver sinusoidal endothelial dysfunction
MASH: metabolic dysfunction–associated steatohepatitis
MMP: metalloproteinase
PH: portal hypertension
sENG: soluble endoglin
GGT: gamma–glutamyltransferase
AST: aspartate aminotransferase
ALT: alanine aminotransferase

## References

[1] Paik, J. M., Henry, L. & Younossi, Z. M. The Global Burden of MASLD in the Past Three Decades. *Liver Int.* **45**, e70127 (2025). 40343732 10.1111/liv.70127

[2] Devarbhavi, H. et al. Global burden of liver disease: 2023 update. *J. Hepatol.* **79**, 516–537 (2023). 36990226 10.1016/j.jhep.2023.03.017

[3] Gan, C. et al. Liver diseases: epidemiology, causes, trends and predictions. *Signal. Transduct. Target. Ther.* **10**, 33 (2025). 39904973 10.1038/s41392-024-02072-z PMC11794951

[4] Pimpin, L. et al. Burden of liver disease in Europe: Epidemiology and analysis of risk factors to identify prevention policies. *J. Hepatol.* **69**, 718–735 (2018). 29777749 10.1016/j.jhep.2018.05.011

[5] Groszmann, R. J. & Wongcharatrawee, S. The hepatic venous pressure gradient: anything worth doing should be done right. *Hepatology* **39**, 280–282 (2004). 14767976 10.1002/hep.20062

[6] Oujo, B., Perez-Barriocanal, F., Bernabeu, C. & Lopez-Novoa, J. M. Membrane and soluble forms of endoglin in preeclampsia. *Curr. Mol. Med.* **13**, 1345–1357 (2013). 23826920 10.2174/15665240113139990058

[7] Aristorena, M. et al. MMP-12, secreted by pro-inflammatory macrophages, targets endoglin in human macrophages and endothelial cells. *Int. J. Mol. Sci.* **20**, 3107 (2019). 10.3390/ijms20123107 PMC6627183 31242676

[8] Hawinkels, L. J. et al. ten Matrix metalloproteinase-14 (MT1-MMP)-mediated endoglin shedding inhibits tumor angiogenesis. *Cancer Res* 70, 4141–4150 (2010). 10.1158/0008-5472.CAN-09-4466 20424116

[9] Valbuena-Diez, A. C. et al. Oxysterol-induced soluble endoglin release and its involvement in hypertension. *Circulation* **126**, 2612–2624 (2012). 23110859 10.1161/CIRCULATIONAHA.112.101261

[10] Leanos-Miranda, A. et al. Soluble Endoglin As a Marker for Preeclampsia, Its Severity, and the Occurrence of Adverse Outcomes. *Hypertension* **74**, 991–997 (2019). 31446801 10.1161/HYPERTENSIONAHA.119.13348

[11] Blann, A. D., Wang, J. M., Wilson, P. B. & Kumar, S. Serum levels of the TGF-beta receptor are increased in atherosclerosis. *Atherosclerosis* **120**, 221–226 (1996). 8645363 10.1016/0021-9150(95)05713-7

[12] Visek, J. et al. Monitoring of up to 15 years effects of lipoprotein apheresis on lipids, biomarkers of inflammation, and soluble endoglin in familial hypercholesterolemia patients. *Orphanet J. Rare Dis.* **16**, 110 (2021). 33640001 10.1186/s13023-021-01749-w PMC7913462

[13] Rathouska, J. et al. Endoglin as a possible marker of atorvastatin treatment benefit in atherosclerosis. *Pharmacol. Res.* **64**, 53–59 (2011). 21440631 10.1016/j.phrs.2011.03.008

[14] Malhotra, R. et al. Circulating angiogenic modulatory factors predict survival and functional class in pulmonary arterial hypertension. *Pulm Circ.* **3**, 369–380 (2013). 24015338 10.4103/2045-8932.110445 PMC3757832

[15] Tomaskova, V. et al. Prognostic value of soluble endoglin in patients with septic shock and severe COVID-19. *Front. Med. (Lausanne)*. **9**, 972040 (2022). 36117974 10.3389/fmed.2022.972040 PMC9470754

[16] Eissazadeh, S. et al. Endoglin and soluble endoglin in liver sinusoidal endothelial dysfunction in vivo. *Biochim. Biophys. Acta Mol. Basis Dis.* **1870**, 166990 (2024). 38110128 10.1016/j.bbadis.2023.166990

[17] Young, M., McLeod, D. S. A. & Richard, K. Nicotine increases hepatocyte transthyretin turnover: A possible mechanism for the protective effect of smoking on preeclampsia? *Mol. Cell. Endocrinol.* **597**, 112446 (2025). 39725350 10.1016/j.mce.2024.112446

[18] Vicen, M. et al. Regulation and role of endoglin in cholesterol-induced endothelial and vascular dysfunction in vivo and in vitro. *FASEB J.* **33**, 6099–6114 (2019). 30753095 10.1096/fj.201802245R

[19] Rathouska, J., Jezkova, K., Nemeckova, I. & Nachtigal, P. Soluble endoglin, hypercholesterolemia and endothelial dysfunction. *Atherosclerosis* **243**, 383–388 (2015). 26520890 10.1016/j.atherosclerosis.2015.10.003

[20] Vitverova, B. et al. Soluble endoglin and hypercholesterolemia aggravate endothelial and vessel wall dysfunction in mouse aorta. *Atherosclerosis* **271**, 15–25 (2018). 29459262 10.1016/j.atherosclerosis.2018.02.008

[21] Varejckova, M. et al. Soluble endoglin modulates the pro-inflammatory mediators NF-kappaB and IL-6 in cultured human endothelial cells. *Life Sci.* **175**, 52–60 (2017). 28336397 10.1016/j.lfs.2017.03.014

[22] Sa, C. I. et al. Labetalol and soluble endoglin aggravate bile acid retention in mice with ethinylestradiol-induced cholestasis. *Front. Pharmacol.* **14**, 1116422 (2023). 36778021 10.3389/fphar.2023.1116422 PMC9909014

[23] Dolezelova, E. et al. High soluble endoglin levels regulate cholesterol homeostasis and bile acids turnover in the liver of transgenic mice. *Life Sci.* **232**, 116643 (2019). 31299237 10.1016/j.lfs.2019.116643

[24] Rath, T. et al. Serum proteome profiling identifies novel and powerful markers of cystic fibrosis liver disease. *PloS one*. **8**, e58955 (2013). 23516586 10.1371/journal.pone.0058955 PMC3597583

[25] Motawi, T. K., Rizk, S. M., Ibrahim, I. A. R. & El-Emady, Y. F. Alterations in circulating angiogenic and anti‐angiogenic factors in type 2 diabetic patients with neuropathy. *Cell Biochem. Funct.* **32**, 155–163 (2014). 23913471 10.1002/cbf.2987

[26] Igreja Sa, I. C. et al. Soluble endoglin as a potential biomarker of nonalcoholic steatohepatitis (NASH) development, participating in aggravation of NASH-related changes in mouse liver. *Int J. Mol. Sci.* **21, **9021 (2020). 10.3390/ijms21239021 PMC7731045 33261044

[27] Prystupa, A. et al. Relationships between serum selenium and zinc concentrations versus profibrotic and proangiogenic cytokines (FGF-19 and endoglin) in patients with alcoholic liver cirrhosis. *Ann. Agric. Environ. Med.* **24**, 544–548 (2017). 28954507 10.26444/aaem/76937

[28] de Franchis, R. et al. Corrigendum to ‘Baveno VII - Renewing consensus in portal hypertension’ [J Hepatol (2022) 959–974]. *J Hepatol* 77, 271 (2022). 10.1016/j.jhep.2022.03.024 35431106

[29] Kaplan, D. E. et al. AASLD Practice Guidance on risk stratification and management of portal hypertension and varices in cirrhosis. *Hepatology* **79**, 1180–1211 (2024). 37870298 10.1097/HEP.0000000000000647

[30] Kulkarni, A. V., Rabiee, A. & Mohanty, A. Management of Portal Hypertension. *J. Clin. Exp. Hepatol.* **12**, 1184–1199 (2022). 35814519 10.1016/j.jceh.2022.03.002 PMC9257868

[31] Garcia-Tsao, G., Abraldes, J. G., Berzigotti, A. & Bosch, J. Portal hypertensive bleeding in cirrhosis: Risk stratification, diagnosis, and management: 2016 practice guidance by the American Association for the study of liver diseases. *Hepatology* **65**, 310–335 (2017). 27786365 10.1002/hep.28906

[32] Vaishnav, M. et al. and. Hepatic Venous Pressure Gradient Predicts Further Decompensation in Cirrhosis Patients with Acute Esophageal Variceal Bleeding. *Diagnostics (Basel)* 13 (2023). 10.3390/diagnostics13142385 PMC10378396 37510129

[33] Suk, K. T. Hepatic venous pressure gradient: clinical use in chronic liver disease. *Clin. Mol. Hepatol.* **20**, 6–14 (2014). 24757653 10.3350/cmh.2014.20.1.6 PMC3992331

[34] Kaitu’u-Lino, T. J. et al. MMP-14 is expressed in preeclamptic placentas and mediates release of soluble endoglin. *Am. J. Pathol.* **180**, 888–894 (2012). 22296769 10.1016/j.ajpath.2011.11.014

[35] Eissazadeh, S. et al. Anti-Endoglin monoclonal antibody prevents the progression of liver sinusoidal endothelial inflammation and fibrosis in MASH. *Life Sci.* **364**, 123428 (2025). 39889923 10.1016/j.lfs.2025.123428

[36] Rathouska, J. U. et al. Soluble endoglin reflects endothelial dysfunction in myocardial infarction patients: a retrospective observational study. *Int. J. Med. Sci.* **22**, 3220–3228 (2025). 40765559 10.7150/ijms.115222 PMC12320647

[37] Lala, V., Zubair, M. & Minter, D. A. Liver Function Tests. In *StatPearls*, Treasure Island (FL) (2025). 29494096

[38] Zhang, Z., Ma, L., Geng, H. & Bian, Y. Effects of Smoking, and Drinking on Serum Gamma-Glutamyl Transferase Levels Using Physical Examination Data: A Cross-Sectional Study in Northwest China. *Int. J. Gen. Med.* **14**, 1301–1309 (2021). 33883928 10.2147/IJGM.S301900 PMC8055286

[39] Yuan, S., Damrauer, S. M. & Larsson, S. C. Alcohol, Liver Disease, and Peripheral Arterial Disease: Epidemiology, Mechanisms, and Clinical Implications. *Arterioscler. Thromb. Vasc Biol.* **45**, 1493–1504 (2025). 40501384 10.1161/ATVBAHA.125.322136 PMC12239223

[40] Toda, N. & Ayajiki, K. Vascular actions of nitric oxide as affected by exposure to alcohol. *Alcohol Alcohol*. **45**, 347–355 (2010). 20522422 10.1093/alcalc/agq028

[41] Mohan, R., Jose, S., Sukumaran, S., John, S. A. S. S., I, M. K. & G., and Curcumin-galactomannosides mitigate alcohol-induced liver damage by inhibiting oxidative stress, hepatic inflammation, and enhance bioavailability on TLR4/MMP events compared to curcumin. *J. Biochem. Mol. Toxicol.* **33**, e22315 (2019). 30793463 10.1002/jbt.22315

[42] Peng, Z. et al. Ecto-5’-nucleotidase (CD73)-mediated extracellular adenosine production plays a critical role in hepatic fibrosis. *Nucleosides Nucleotides Nucleic Acids*. **27**, 821–824 (2008). 18600546 10.1080/15257770802146403

[43] McConnell, M. J. & Iwakiri, Y. Portal Hypertension in Alcohol-Associated Hepatitis. *Curr. Hepatol. Rep.* **22**, 67–73 (2023). 37214274 10.1007/s11901-023-00601-y PMC10075503

[44] Torp, N., Israelsen, M., Thiele, M., Rinella, M. E. & Krag, A. Phosphatidylethanol in steatotic liver disease. *J. Hepatol.* **83**, 1189–1203 (2025). 40759197 10.1016/j.jhep.2025.07.019

[45] Kummetat, J. L., Leonhard, A., Manthey, J., Speerforck, S. & Schomerus, G. Understanding the Association between Alcohol Stigma and Alcohol Consumption within Europe: A Cross-Sectional Exploratory Study. *Eur. Addict. Res.* **28**, 446–454 (2022). 36088900 10.1159/000526200

